# Bezlotoxumab for Prevention of Recurrent *Clostridium difficile* Infection in Patients at Increased Risk for Recurrence

**DOI:** 10.1093/cid/ciy171

**Published:** 2018-03-10

**Authors:** Dale N Gerding, Ciaran P Kelly, Galia Rahav, Christine Lee, Erik R Dubberke, Princy N Kumar, Bruce Yacyshyn, Dina Kao, Karen Eves, Misoo C Ellison, Mary E Hanson, Dalya Guris, Mary Beth Dorr

**Affiliations:** 1 Department of Veterans Affairs, Edward Hines Jr Veterans Affairs Hospital, Illinois; 2 Gastroenterology Division, Beth Israel Deaconess Medical Center and Harvard Medical School, Boston, Massachusetts; 3 Infectious Diseases, Sheba Medical Center, Tel Hashomer, and Sackler School of Medicine, Tel-Aviv University, Israel; 4 Department of Pathology and Laboratory Medicine, University of British Columbia, Vancouver; 5 McMaster University, Hamilton, Ontario, Canada; 6 Division of Infectious Diseases, Washington University School of Medicine, St Louis, Missouri; 7 Division of Infectious Diseases and Travel Medicine, Georgetown University School of Medicine, Washington, D.C; 8 Division of Digestive Diseases, University of Cincinnati, Ohio; 9 Department of Medicine, Division of Gastroenterology, University of Alberta, Edmonton, Canada; 10 Clinical Research, Infectious Diseases, Kenilworth, New Jersey; 11 Late Development Statistics, Biostatistics, Kenilworth, New Jersey; 12 Global Scientific and Medical Publications, Infectious Diseases, Merck & Co, Inc, Kenilworth, New Jersey

**Keywords:** *C. difficile* infection, CDI; recurrence, bezlotoxumab

## Abstract

**Background:**

Bezlotoxumab is a human monoclonal antibody against *Clostridium difficile* toxin B indicated to prevent *C. difficile* infection (CDI) recurrence (rCDI) in adults at high risk for rCDI. This post hoc analysis of pooled monocolonal antibodies for *C.difficile* therapy (MODIFY) I/II data assessed bezlotoxumab efficacy in participants with characteristics associated with increased risk for rCDI.

**Methods:**

The analysis population was the modified intent-to-treat population who received bezlotoxumab or placebo (n = 1554) by risk factors for rCDI that were prespecified in the statistical analysis plan: age ≥65 years, history of CDI, compromised immunity, severe CDI, and ribotype 027/078/244. The proportion of participants with rCDI in 12 weeks, fecal microbiota transplant procedures, 30-day all cause and CDI-associated hospital readmissions, and mortality at 30 and 90 days after randomization were presented.

**Results:**

The majority of enrolled participants (75.6%) had ≥1 risk factor; these participants were older and a higher proportion had comorbidities compared with participants with no risk factors. The proportion of placebo participants who experienced rCDI exceeded 30% for each risk factor compared with 20.9% among those without a risk factor, and the rCDI rate increased with the number of risk factors (1 risk factor: 31.3%; ≥3 risk factors: 46.1%). Bezlotoxumab reduced rCDI, fecal microbiota transplants, and CDI-associated 30-day readmissions in participants with risk factors for rCDI.

**Conclusions:**

The risk factors prespecified in the MODIFY statistical analysis plan are appropriate to identify patients at high risk for rCDI. While participants with ≥3 risk factors had the greatest reduction of rCDI with bezlotoxumab, those with 1 or 2 risk factors may also benefit.

**Clinical Trials Registration:**

NCT01241552 (MODIFY I) and NCT01513239 (MODIFY II).

## Teaser

Subgroup analyses from MODIFY confirmed that prior *Clostridium difficile* infection (CDI), age ≥65 years, infection with 027/078/244 strain, compromised immunity, and severe CDI are risk factors for recurrent CDI. Bezlotoxumab reduced CDI recurrence in participants with ≥1 risk factor compared with placebo.

*Clostridium difficile* is a gram-positive, anaerobic, spore- forming bacillus, ubiquitous in nature and able to survive long periods in the environment. *Clostridium difficile* infection (CDI) occurs in the gastrointestinal tract of individuals whose normal gut microbiota has been disrupted, usually by prior antimicrobial use, leading to loss of colonization resistance and opportunistic infection with *C. difficile* [1]. Treatment requires specific antibiotic therapy to eliminate *C. difficile* from the colon; however, after completing initial antibiotic therapy with metronidazole or vancomycin, approximately 25% of patients have recurrent CDI (rCDI) [2, 3], and approximately 40% with a first recurrence experience a second recurrence [4]. Certain host or pathogen factors have been associated with an increased risk of rCDI or CDI-related adverse outcomes: age ≥65 years [5, 6], compromised immunity [7], severe CDI [8, 9], prior CDI episode(s) [4], and infection with the BI/NAP1/027 strain [5, 10–12]. As the incidence of rCDI is rising, there is an unmet need for therapies to prevent rCDI [13].

In 2 global, phase 3 trials (monocolonal antibodies for C. difficile therapy [MODIFY] I and MODIFY II), bezlotoxumab, a human monoclonal antibody against *C. difficile* toxin B, demonstrated significant reductions in CDI recurrence compared with placesbo (17% vs 28% in MODIFY I and 16% vs 26% in MODIFY II; *P* < .001) in adults receiving antibiotic treatment for primary CDI or rCDI [14]. To identify the population that may benefit most from bezlotoxumab treatment, the current analysis pooled data from the MODIFY studies to assess the efficacy of bezlotoxumab in participants with the characteristics listed above that are associated with increased risk for rCDI.

## METHODS

MODIFY I (NCT01241552) and II (NCT01513239) were independent, randomized, double-blind, placebo-controlled, multicenter phase 3 trials with nearly identical design, conducted from November 2011 through May 2015 at 322 sites in 30 countries. The protocols and all amendments were approved by the institutional review board or independent ethics committee at each study center. Each study was conducted in accordance with Good Clinical Practice Guidelines and the Declaration of Helsinki. Written informed consent was obtained before study procedures were performed.

Adults with primary or rCDI receiving antibacterial treatment (determined by the treating physician) for CDI were enrolled. Eligibility criteria were previously described [14]. CDI was defined as diarrhea (≥3 unformed bowel movements in 24 hours) associated with a positive stool test for toxigenic *C. difficile* or its toxin(s). Participants included in this subgroup analysis received either 1 dose of bezlotoxumab 10 mg/kg or placebo (0.9% saline) during antibacterial drug treatment for CDI. Randomization was stratified by oral antibacterial treatment (metronidazole, vancomycin, or fidaxomicin) and hospitalization status (inpatient or outpatient). The number of unformed bowel movements was recorded by participants daily through 12 weeks and new episodes of diarrhea were monitored via scheduled phone contacts between visits.

### Population, Endpoints, and Statistical Methods

The analysis population was the modified intent-to-treat (mITT) population (defined as all randomly assigned participants who received study infusion, had a positive toxigenic *C. difficile*–positive stool test, and received standard-of-care [SOC] therapy within 1 day of receiving treatment) in the pooled dataset from MODIFY I and MODIFY II, who received bezlotoxumab or placebo, and was further categorized based on known risk factors for rCDI or CDI-related adverse outcomes that had been prespecified at the time of protocol development in the statistical analysis plan: age ≥65 years, history of CDI in the previous 6 months, compromised immunity, severe CDI (Zar score ≥2 points) at time of randomization, and isolation of a strain associated with poor outcomes (ribotypes 027, 078, or 244). Use of concomitant systemic antibiotics, although a known risk factor for rCDI [15], was not prespecified as a risk factor for subgroup analysis because only risk factors present at the time of randomization were included.

Endpoints included proportion of participants who achieved initial clinical cure, where initial clinical cure is defined as no diarrhea for 2 consecutive days after completion of antibiotic therapy administered for ≤16 calendar days; proportion of participants with rCDI, where rCDI is defined as the development of a new episode of diarrhea associated with a positive stool test for toxigenic *C. difficile* within 12 weeks following study medication infusion assessed in mITT participants who achieved initial clinical cure (clinical cure population); proportion of participants who received a fecal microbiota transplant (FMT), proportion of participants who experienced 30-day all cause and CDI-associated hospital readmissions as defined previously [16] in mITT participants who were in a healthcare facility at the time of randomization; and proportion of participants who had died at 30 and 60 days after randomization.

Observed rates for rCDI along with differences between the bezlotoxumab and placebo groups and their 95% confidence intervals (CIs) were presented by prespecified risk factor subgroup and by combinations of risk factors. The 95% CIs were obtained using the Miettinen and Nurminen method [17]. The nonparametric Kaplan-Meier method was used to estimate the distribution of time to CDI recurrence for each subgroup within each treatment group. Observed rates were presented for those requiring FMT during the 12-week follow-up period, 30-day all-cause and CDI-associated hospital readmissions, and mortality at 30 and 60 days after randomization.

## RESULTS

### Study Population

There were 1554 participants (bezlotoxumab: 781; placebo: 773) included in the mITT population in both studies. The majority of participants (75.6% [1175/1554]: 592 bezlotoxumab, 583 placebo) had at least 1 prespecified risk factor for rCDI or CDI-related adverse outcomes: Approximately 36% had a single risk factor, approximately 27% had 2 risk factors, and approximately 12% had ≥3 risk factors (Table 1). In the group of participants with at least 1 risk factor, 497 (84.0%) and 482 (82.7%) participants remained in the study through week 12 in the bezlotoxumab and placebo groups, respectively. A higher proportion of participants with no risk factors completed the study (91.5% bezlotoxumab, 86.8% placebo) compared with participants with at least 1 risk factor. Reasons for discontinuation by treatment group and by risk category are summarized in Supplementary Table 1. The proportion of participants who prematurely discontinued the study due to death was higher in participants with at least 1 risk factor (7.8% bezlotoxumab, 8.6% placebo) compared with the proportion with no risk factors (3.2% for both bezlotoxumab and placebo).

**Table 1.** Baseline Demographics, Clinical Characteristics, and Predefined Risk Factors, Modified Intent-to-Treat Population

Characteristic	Bezlotoxumab (N = 781)		Placebo (N = 773)	
	No Risk Factors	≥1 Risk Factor	No Risk Factors	≥1 Risk Factor
Demographics	n = 189	n = 592	n = 190	n = 583
Age, y				
Mean (SD)	46.6 (12.8)	66.7 (16.7)	48.2 (13.2)	68.5 (15.6)
Median	51	69	52	71
Range	20–64	18–100	19–64	18–98
18–49	87 (46.0)	87 (14.7)	81 (42.6)	72 (12.3)
50–64	102 (54.0)	115 (19.4)	109 (57.4)	106 (18.2)
65–79	NA	250 (42.2)	NA	253 (43.4)
≥80	NA	140 (23.6)	NA	152 (26.1)
Female sex	117 (61.9)	325 (54.9)	118 (62.1)	331 (56.8)
SOC antibiotic				
Metronidazole	127 (67.2)	252 (42.6)	124 (65.3)	250 (42.9)
Vancomycin	59 (31.2)	313 (52.9)	63 (33.2)	310 (53.2)
Fidaxomicin	3 (1.6)	27 (4.6)	3 (1.6)	23 (3.9)
Clinical characteristics				
Age ≥65 y^a^	NA	390 (65.9)	NA	405 (69.5)
Primary CDI	146 (77.2)	278 (47.0)	145 (76.3)	255 (43.7)
≥1 CDI episodes in past 6 mo^a^	NA	216 (36.5)	NA	219 (37.6)
1 previous CDI episode ever	6 (3.2)	144 (24.3)	6 (3.2)	126 (21.6)
≥2 previous CDI episodes ever	4 (2.1)	96 (16.5)	4 (2.2)	122 (21.4)
Severe CDI (Zar score ≥2)^a,b^	NA	122 (20.6)	NA	125 (21.4)
Immunocompromised^a,c^	NA	178 (30.1)	NA	153 (26.2)
Inpatient at time of randomization	95 (50.3)	435 (73.5)	98 (51.6)	422 (72.4)
Antibiotic use^d^ during SOC	45 (23.8)	201 (34.0)	58 (30.5)	218 (37.4)
Antibiotic use^d^ after SOC	38 (20.1)	208 (35.1)	45 (23.7)	179 (30.7)
Charlson index ≥3	48 (25.4)	271 (45.8)	43 (22.6)	260 (44.6)
Renal impairment^e^	12 (6.3)	111 (18.8)	20 (10.5)	90 (15.4)
Hepatic impairment^f^	12 (6.3)	37 (6.3)	9 (4.7)	35 (6.0)
Albumin <2.5 g/dL	13 (6.9)	88 (14.9)	9 (4.7)	94 (16.1)
*Clostridium difficile* strain^g^				
Participants with a positive culture	112 (59.3)	378 (63.9)	114 (60.0)	372 (63.8)
Ribotype 027, 078, or 244^a^	NA	102 (27.0)	NA	115 (30.9)
Ribotype 027	NA	89 (23.5)	NA	100 (26.9)
No. of prespecified risk factors				
0	189 (24.2)		190 (24.6)	
1	283 (36.2)		274 (35.4)	
2	220 (28.2)		208 (26.9)	
3	73 (9.3)		82 (10.6)	
4	14 (1.8)		14 (2.0)	
5	2 (0.3)		5 (0.6)	

Data are presented as No. (%) unless otherwise indicated.

Abbreviations: CDI, *Clostridium difficile* infection; NA, not analyzed; SD, standard deviation; SOC, standard of care.

^a^Prespecified risk factor.

^b^Based on the following: (1) age >60 years (1 point); (2) body temperature >38.3°C (>101°F) (1 point); (3) albumin level <2.5 g/dL (1 point); (4) peripheral white blood cell count >15000 cells/µL within 48 hours (1 point); (5) endoscopic evidence of pseudomembranous colitis (2 points); and (6) treatment in an intensive care unit (2 points).

^c^Defined on the basis of a participant’s medical history or use of immunosuppressive therapy.

^d^Systemic antibiotic other than SOC antibiotic given to treat CDI.

^e^Renal impairment defined as serum creatinine ≥1.5 mg/dL.

^f^Hepatic impairment defined by ≥2 of the following: (1) albumin ≤3.1 g/dL; (2) alanine aminotransferase ≥2 times the upper limit of normal (ULN); (3) total bilirubin ≥1.3 times the ULN; or (4) mild, moderate, or severe liver disease (as reported on the Charlson index).

^g^Denominator is participants in the modified intent-to-treat population with a positive culture.

Demographic and clinical characteristics were similar between the bezlotoxumab and placebo groups when comparing within each of the 2 risk categories (Table 1). The participants with at least 1 risk factor were older and a higher percentage had a Charlson index ≥3, had renal impairment, had hypoalbuminemia, and received vancomycin or fidaxomicin as the SOC antibiotic (Table 1) compared with the group of participants with no risk factors.

### Initial Clinical Cure

Treatment with bezlotoxumab did not impact the proportion of participants with initial clinical cure of the episode under treatment with SOC antibiotics. Initial clinical cure rates were similar in the bezlotoxumab and placebo groups in participants with at least 1 risk factor (79.6% vs 80.3%, respectively; difference, –0.7 [95% CI, –5.3 to 3.9]) and in the participants with no risk factors (81.5% vs 80.5%, respectively; difference, 1.0 [95% CI, –7.0 to 8.9]).

### CDI Recurrence

**Figure 1.**
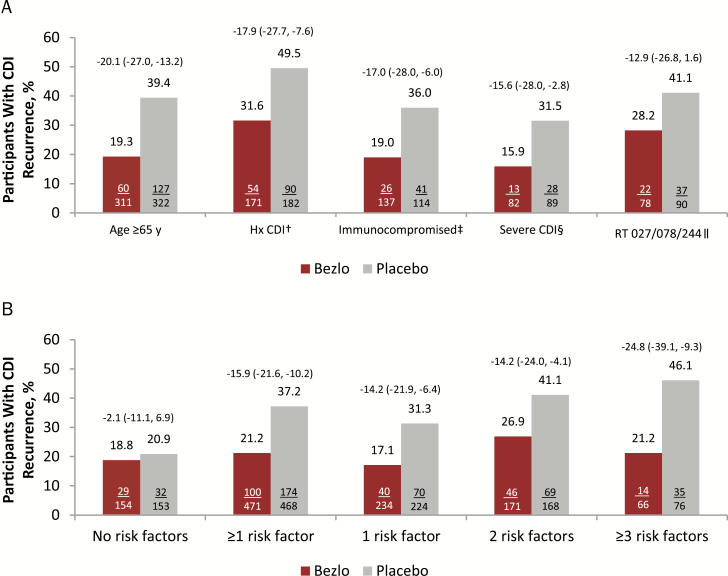
*A*, Proportion of participants with *Clostridium difficile* infection (CDI) recurrence, by prespecified risk factor. Each subgroup includes all participants with the risk factor (ie, those with only 1 risk factor and those with the specified risk factor and ≥1 additional risk factor). *B*, Proportion of participants with CDI recurrence, by number of prespecified risk factors (clinical cure population). Difference and 95% confidence intervals, shown above bars, were based on the Miettinen and Nurminen method. Prespecified risk factors include age ≥65 years, history of CDI in the previous 6 months, immunocompromised, severe CDI, and having a strain associated with poor outcomes of CDI (ribotype 027, 078, or 244). ^†^CDI history in the previous 6 months. ^‡^Defined on the basis of a subject’s medical history or use of immunosuppressive therapy. ^§^Based on the Zar score, scored as (1) age >60 years (1 point); (2) body temperature >38.3°C (>101°F) (1 point); (3) albumin level <2.5 g/dL (1 point); (4) peripheral white blood cell count >15000 cells/µL within 48 hours (1 point); (5) endoscopic evidence of pseudomembranous colitis (2 points); and (6) treatment in an intensive care unit (2 points). ^॥^Denominator is participants in the modified intent-to-treat population with a positive culture. Abbreviations: Bezlo, bezlotoxumab; CDI, *Clostridium difficile* infection; Hx, history; RT, ribotype.

Among participants who received placebo and achieved initial clinical cure, the proportion of participants who experienced rCDI during the 12-week follow-up period exceeded 30% for each of the 5 prespecified risk factors (Figure 1A). The proportion of participants who experienced a recurrence in the placebo group was 20.9% among those without a risk factor and increased with the number of risk factors (Figure 1B), 31.3% for participants with 1 risk factor, and 46.1% in participants with at least 3 risk factors.

Bezlotoxumab reduced the rate of rCDI compared with placebo among participants with each of the 5 prespecified risk factors, and the 95% CIs excluded 0 for all comparisons except the ribotype 027/078/244 subgroup (Figure 1A). Among participants who had ≥1 risk factor, treatment with bezlotoxumab reduced CDI recurrences compared with placebo (21.2% vs 37.2%, respectively; difference, –15.9 [95% CI, –21.6 to –10.2]; Figure 1B). The absolute reduction was –14.2%, –14.2%, and –24.8% (relative reduction: –45.3%, –34.5%, and –53.9%) for those with 1, 2, and ≥3 risk factors, respectively (95% CIs excluded 0 for all comparisons; Figure 1B). In contrast, among participants with no risk factors, the proportion of participants with CDI recurrences was similar between treatment groups (18.8% bezlotoxumab vs 20.9% placebo; difference, –2.1 [95% CI, –11.1 to 6.9]; Figure 1B).

**Figure 2.**
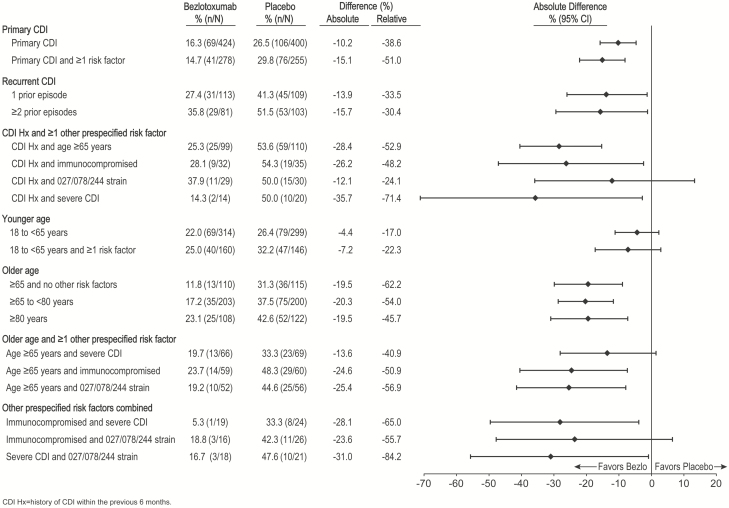
*Clostridium difficile* infection (CDI) recurrence rates by risk factor subgroup (clinical cure population). Unless otherwise specified, each subgroup includes all patients with the risk factor(s) (ie, those with only the specified risk factor[s] and those with the specified risk factor[s] and ≥1 additional risk factor). Abbreviations: CDI Hx, *Clostridium difficile* infection history in the previous 6 months; CI, confidence interval.

The proportion of participants experiencing rCDI in clinically relevant subpopulations is shown in Figure 2. In the placebo-treated participants with a single risk factor, the rate of rCDI ranged from 29.8% in participants experiencing primary CDI who had at least 1 risk factor, to 54.3% in participants who had at least 1 prior episode of CDI and were immunocompromised.

In all subgroups assessed, including participants with primary CDI (with or without any prespecified risk factors), rCDI rates were lower in participants treated with bezlotoxumab compared with those treated with placebo (Figure 2). The absolute difference in the rate of rCDI between the bezlotoxumab and placebo groups was highest in participants who had both a history of CDI and severe CDI (–35.7% [95% CI, –60.5% to –2.8%]) and was lowest in patients who were <65 years of age (–4.4% [95% CI, –11.2% to 2.3%]). Of note, the 95% CIs crossed 0 for some of these subgroups due to small differences (eg, younger age) or due to the limited size of the subgroup (eg, history of CDI and infected with ribotype 027/078/244).

**Figure 3.**
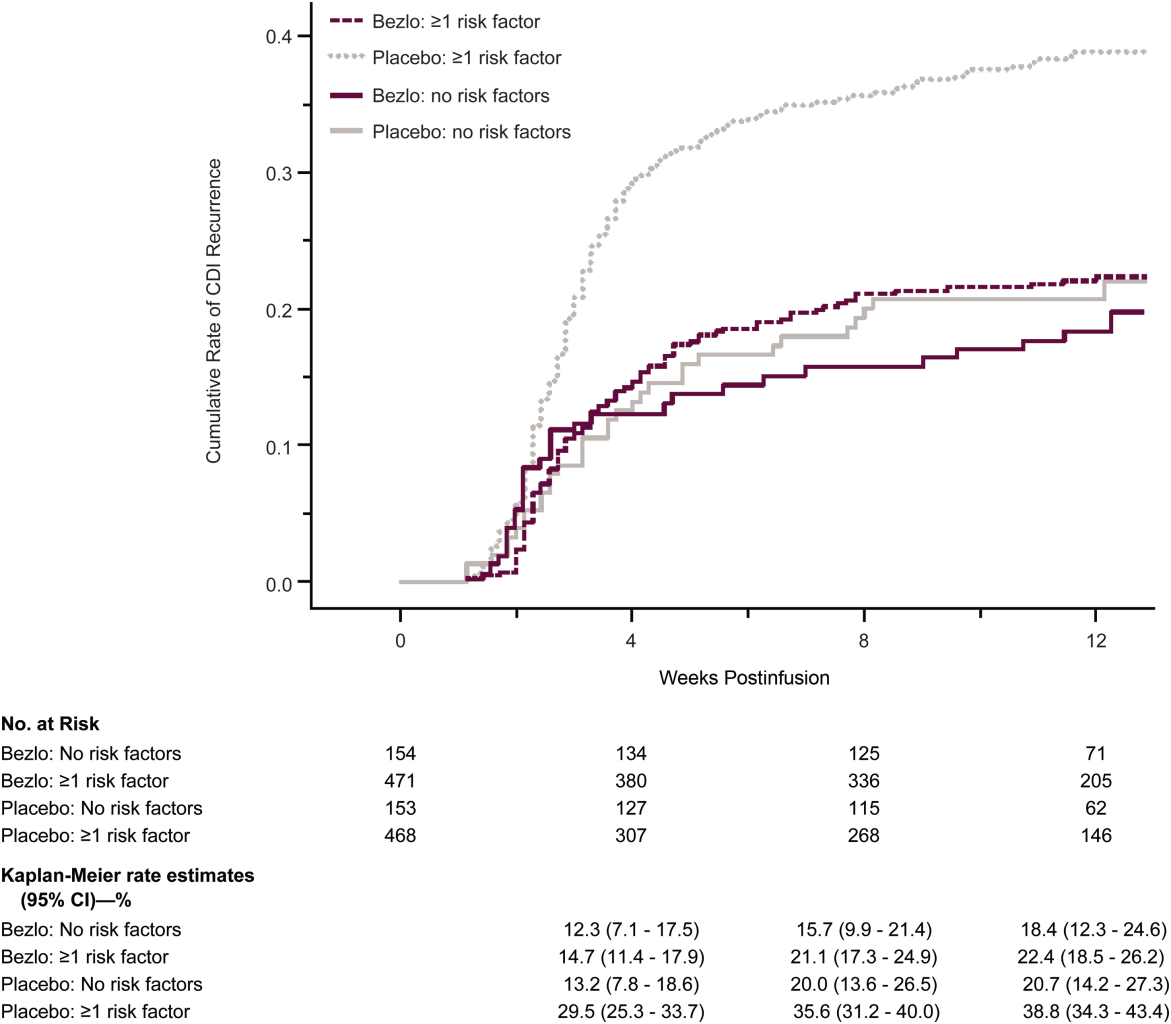
Kaplan-Meier plot of time to *Clostridium difficile* infection (CDI) recurrence over 12 weeks of follow-up by high-risk-factor subgroups (modified intent-to-treat population). The start date of CDI recurrence was the first date of the new episode of diarrhea. For subjects who were lost to follow-up prior to a CDI recurrence, time to event was right-censored at the date of the last stool record. Participants who completed the 12-week follow-up period without documented CDI recurrence were censored at the date of the last completed stool record. For participants who failed to achieve a clinical cure for the baseline CDI episode, time to event was right-censored at the date of infusion of study medication (day 1). Abbreviations: Bezlo, bezlotoxumab; CDI, *Clostridium difficile* infection; CI, confidence interval.

Most CDI recurrences occurred within 4 weeks of study infusion (range, 63%–78% across subgroups; Figure 3). There were differences in the rate of rCDI at week 4 between participants with ≥1 risk factor who received placebo compared with participants with ≥1 risk factor who received bezlotoxumab, and these differences were maintained through week 12. In participants with no risk factors who received either placebo or bezlotoxumab, the time-to-event curve was similar to the curve for the bezlotoxumab group with ≥1 risk factor (Figure 3).

### Other Outcomes

The proportion of participants who (1) had an FMT during the 90-day follow-up period, (2) were readmitted to the hospital within 30 days of discharge, and (3) died within 30 or 90 days of study entry was higher in participants with at least 1 risk factor for rCDI compared with those with no risk factors (Table 2). Among participants in the placebo group, there were more FMTs and more CDI-associated 30-day hospital readmissions compared with bezlotoxumab participants in both risk factor categories (Table 2).

**Table 2.** Other Outcomes

Outcome	Bezlotoxumab		Placebo	
	No Risk Factors	≥1 Risk Factor	No Risk Factors	≥1 Risk Factor
FMT during follow-up^a^	n = 189	n = 592	n = 190	n = 583
	0 (0.0)	7 (1.2)	5 (2.6)	18 (3.1)
30-day readmissions^b^	n = 95	n = 435	n = 98	n = 422
All-cause	20 (21.1)	103 (23.7)	20 (20.4)	120 (28.4)
CDI-associated	2 (2.1)	25 (5.7)	4 (4.1)	54 (12.8)
Mortality^c^	n = 189	n = 597	n = 192	n = 589
30-day	2 (1.1)	25 (4.2)	3 (1.6)	24 (4.1)
90-day	6 (3.2)	48 (8.0)	6 (3.1)	53 (9.0)

Abbreviations: CDI, *Clostridium difficile* infection; FMT, fecal microbiota transplant.

^a^Modified intent-to-treat (mITT) population.

^b^mITT population who were inpatients at the time of randomization.

^c^All patients as treated population.

## DISCUSSION

Individual characteristics such as age ≥65 years [5, 18], compromised immunity [7], severe CDI [8, 9], history of CDI [4], and infection with the BI/NAP1/027 strain [5, 10–12] have been shown to increase a patient’s risk for rCDI and/or CDI-related adverse outcomes. However, previous studies have not investigated the impact of multiple risk factors on these outcomes. The current analysis included data from 2 large, prospective, randomized, placebo-controlled trials that enrolled participants with multiple risk factors [14]. Approximately 75% of the participants enrolled in the MODIFY trials had at least 1 of 5 prespecified risk factors, and 40% had at least 2 risk factors. Based on the data from the group of participants who received placebo, this post hoc analysis confirmed that presence of at least 1 of the 5 prespecified risk factors increased the risk for rCDI, 30-day and 90-day mortality, 30-day CDI-associated hospital readmissions, and need for FMT compared with having none of these 5 risk factors, with the exception of hypervirulent strains. Furthermore, as the number of risk factors increased, the proportion of placebo participants with rCDI also increased and was greatest in participants with at least 3 risk factors.

The most frequently cited risk factor for rCDI is having had a previous episode. The rate of rCDI in participants receiving placebo in the MODIFY trials who were experiencing their first episode was 26.5%. The rate climbed to 41.3% in participants who had 1 prior episode and was 51.5% in participants with ≥2 prior episodes. These rates are comparable with previously reported rates [4]. Of note, at least 50% of placebo-treated participants who had both a recent history of CDI and at least 1 other risk factor experienced rCDI within 12 weeks of infusion. While participants with primary CDI had lower rates of rCDI, the rate was approximately 30% in the primary CDI subgroup that had at least 1 risk factor, suggesting that there are some patients with primary CDI who may be considered to be at high risk for rCDI.

Older age (≥65 years) has been characterized as a risk factor in almost all studies reporting on risk factors for rCDI. While it is generally assumed that the higher rate in older patients is likely because they have other risk factors that contribute to the higher rate of rCDI compared with younger patients, in this analysis, almost one-third of placebo-treated participants with age ≥65 years as their sole risk factor experienced rCDI. As expected, the proportion of older placebo participants who experienced rCDI and had an additional risk factor was higher than those ≥65 years as their only risk factor (range, 33.3%–48.3%). A lower rate of rCDI was observed in younger participants (age <65 years, 26.4%). However, the rate exceeded 30% in the subgroup of younger participants who had at least 1 of the 4 other risk factors.

A major strength of this analysis was that it identified patients who are likely to benefit from treatment with bezlotoxumab. In participants with at least 1 risk factor, treatment with bezlotoxumab reduced the proportion of participants with rCDI over a 12-week period compared with placebo (absolute reduction 16%, relative reduction 43%); a larger reduction was observed in participants with ≥3 risk factors (absolute reduction 24.8%, relative reduction 54%).

The proportion of participants with no risk factors who experienced rCDI in the MODIFY trials was relatively low in both treatment groups (~20%). In general, participants without 1 of these known risk factors for rCDI at the time of diagnosis are not likely to benefit from bezlotoxumab. It is important to note, however, that participants with primary CDI and without one of the prespecified risk factors may have other risk factors such as renal impairment [19], inflammatory bowel disease [20], or concomitant antibiotic use [21] that were not included in this analysis and could also benefit from bezlotoxumab therapy for prevention of a future recurrence. Ongoing analyses may identify other at-risk populations that will benefit from bezlotoxumab treatment.

Results for other post hoc endpoints are supportive of the benefit of bezlotoxumab in patients at high risk for rCDI or CDI-related adverse outcomes. Fewer bezlotoxumab-treated participants received an FMT during the 12-week follow-up period compared with the placebo-treated participants. While this observation is encouraging, it should be noted that FMT procedures were performed at only 17 trial sites, which reflects that the study was not limited to sites that have access to this procedure; additional research is needed to confirm these results. Bezlotoxumab also reduced the number of CDI-related hospital readmissions in participants with at least 1 risk factor compared with the placebo group (difference, –7.1%). These results are consistent with a previous analysis of bezlotoxumab data demonstrating a reduction in 30-day CDI-associated hospital readmissions in those aged ≥65 years and with severe CDI [16]. Although the presence of risk factors increased 30- and 90-day mortality rates, the relatively low rates observed in this study vs those observed in observational studies of patients with CDI [22, 23] may be in part due to the study entry requirement for participants to survive at least 72 hours, potentially biasing the results to the null when assessing differences in mortality.

There were limitations to this analysis. Although the clinical characteristics that were prespecified in the protocol are known risk factors for rCDI or CDI-related adverse outcomes, there are other risk factors for rCDI that were not included. Results may be different if other risk factors are considered. Although there were a large number of participants with at least 1 risk factor included (n = 1175), and the baseline characteristics among these participants across treatment groups were well balanced, many of the analyses presented here were conducted post hoc and the study was not powered for statistical significance for subgroup analyses. Therefore, the results should be considered preliminary.

In conclusion, these results demonstrate that the risk factors prespecified in the MODIFY statistical analysis plan are appropriate to identify patients who are at high risk for rCDI. While participants with at least 3 risk factors appear to have the greatest risk reduction with bezlotoxumab, those with only 1 of the risk factors may also benefit from bezlotoxumab. These observations may help direct clinicians to patients who are most likely to benefit from bezlotoxumab.

## Supplementary Data

Supplementary materials are available at *Clinical Infectious Diseases* online. Consisting of data provided by the authors to benefit the reader, the posted materials are not copyedited and are the sole responsibility of the authors, so questions or comments should be addressed to the corresponding author.

Supplementary Materials

## Notes

***Acknowledgments.*** Carol Zecca of Merck & Co, Inc, Kenilworth, New Jersey, provided editorial and submission support; Alison Pedley of Merck & Co, Inc, Kenilworth, New Jersey, provided statistical support.

***Financial support.*** This work was supported by Merck & Co, Inc, Kenilworth, New Jersey.

***Potential conflicts of interest.*** D. N. G. has received fees for serving on advisory boards from Actelion Pharmaceuticals, Rebiotix, Summit Pharmaceuticals, and Merck; has received consulting fees from Da Volterra, Pfizer, Sanofi Pasteur, and MGB Pharma; has received a research grant from Seres Therapeutics; and has issued patents related to the treatment and prevention of CDI (US patent number 6635260; European Union patent number 0952773; Canada patent number 2232001). C. P. K. has served as a scientific advisor for Facile Therapeutics, Summit (Oxford), Synthetic Biologics, Actelion, First Light Diagnostics, Finch, GlaxoSmithKline, Merck, Seres Therapeutics, CellImmune, Core Pharma, Takeda, Innovate, Immunogenex, and Vedanta; has received lecture fees from Merck and Seres Therapeutics; and has received grant support from the National Institutes of Health, Institut Mérieux, Aptalis, and Merck. G. R. has received fees for lectures and advisory board membership from Pfizer and Merck Sharp & Dohme. C. L. has received grant support from Rebiotix, Merck, and Actelion Pharmaceuticals and fees for serving on advisory boards from Rebiotix and Summit Pharmaceuticals. E. R. D. is an investigator on behalf of Merck & Co, Inc and Rebiotix; has received grants from Sanofi Pasteur; and is a consultant for Merck & Co, Inc, GSK, Velneva, Rebiotix, and Sanofi Pasteur. P. N. K. has served on advisory boards for Gilead, Johnson & Johnson, GSK, Theratechnolgies and Gilead; and holds stock in Gilead, Johnson & Johnson, Pfizer, Merck, and GSK. B. Y. has served on speaker’s bureaus for Allergan, Merck, and UCB; is an advisory board member for Summit and a data and safety monitoring board member for Seres; has received grant support and travel support from Merck; and has served as a consultant for Aerpio. D. K. has received grant support from Rebiotix. K. E., M. C. E., M. E. H., D. G., and M. B. D. are employees of Merck & Co, Inc, Kenilworth, New Jersey, and may own stock or hold stock options in the company. All authors have submitted the ICMJE Form for Disclosure of Potential Conflicts of Interest. Conflicts that the editors consider relevant to the content of the manuscript have been disclosed.
