# Supplementary material for: Bezlotoxumab for Prevention of Recurrent Clostridium difficile Infection in Patients at Increased Risk for Recurrence
Source: Clin Infect Dis. 2018 Mar 10;67(5):649–56. doi: 10.1093/cid/ciy171 (PMC6093994; doi:10.1093/cid/ciy171)
Supplement: Supplementary Materials [file ciy171_suppl_supplementary_materials.docx]

**Supplementary Materials**

Supplementary Table 1. Disposition of Participants by Risk Factor Category and Treatment Group (mITT Population)

|  | Bezlotoxumab | | | | Placebo | | | |
| --- | --- | --- | --- | --- | --- | --- | --- | --- |
|  | No risk factors | | ≥1 risk factor | | No risk factors | | ≥1 risk factor | |
|  | n | % | n | % | n | % | n | % |
| Participants in population | 189 |  | 592 |  | 190 |  | 583 |  |
| **Main Study Disposition** |  |  |  |  |  |  |  |  |
| Completed | 173 | 91.5 | 497 | 84.0 | 165 | 86.8 | 482 | 82.7 |
| Discontinued | 16 | 8.5 | 95 | 16.0 | 25 | 13.2 | 101 | 17.3 |
| Adverse Event | 0 | 0.0 | 1 | 0.2 | 0 | 0.0 | 2 | 0.3 |
| Death | 6 | 3.2 | 46 | 7.8 | 6 | 3.2 | 50 | 8.6 |
| Lost To Follow-Up | 6 | 3.2 | 15 | 2.5 | 9 | 4.7 | 13 | 2.2 |
| Physician Decision | 0 | 0.0 | 3 | 0.5 | 1 | 0.5 | 2 | 0.3 |
| Progressive Disease | 0 | 0.0 | 0 | 0.0 | 1 | 0.5 | 1 | 0.2 |
| Protocol Violation | 0 | 0.0 | 0 | 0.0 | 1 | 0.5 | 1 | 0.2 |
| Withdrawal By Participant | 4 | 2.1 | 30 | 5.1 | 7 | 3.7 | 32 | 5.5 |
| Each participant is counted once for Trial Disposition. | | | | | | | | |
